# A New Friedelane Type Triterpene from *Euonymus hederaceus*

**DOI:** 10.3390/molecules14072650

**Published:** 2009-07-17

**Authors:** Cui-Rong Sun, He-Jiao Hu, Run-Sheng Xu, Jie-Hong Yang, Hai-Tong Wan

**Affiliations:** 1 Department of Chemistry, Zhejiang University, Hangzhou 310027, China; 2 Institute of Cardio-Cerbrovascular Disease, Zhejiang Chinese Medical University, Hangzhou 310053, China; E-mail: yangjiehong@zjtcm.net (J-H.Y.)

**Keywords:** *Euonymus hederaceus*, new friedelane triterpene, 28-hydroxyfriedelan- 3-one-29-oic acid, structure elucidation

## Abstract

*Euonymus hederaceus* is distributed widely in the south of China; its stems and leaves have been used as folk medicines to treat many diseases such as renal deficiency and chronic diarrhea, traumatic injury, and abnormal menstruation. Chemical investigation of the leaves and stems of *Euonymus hederaceus* resulted in the isolation for the first time and characterization of a new friedelane type triterpene with a molecular mass of 472 and molecular formula of C_30_H_48_O_4_ by high resolution mass spectrometry. The ^1^H-NMR ^13^C-NMR and DEPT135^0^ spectra matched the characteristic data of the proposed triterpene skeleton. The compound was finally identified as 28-hydroxyfriedelan-3-one-29-oic acid on the basis of spectroscopic evidence, including two dimensional nuclear magnetic resonance as well as its IR spectrum.

## Introduction

Celastraceae plants have been the subject of continued and growing interest due to the range of biological activities shown by many members of this family. Pharmaceutical studies and clinical practice have demonstrated that their sesquiterpenes and triterpenes possess notable antibacterial, anti-tumor, insect antifeedant and cytotoxic activities. More than 100 compounds have been isolated and purified from three species, including *Celastrus hypoleucus* (Oliv.) Warb [1], *Celastrus hypoleucus* [2], and *Microtropis triflora* Merr [3] belonging to the *Celastraceae* family. The structures were determined by IR, UV, MS, and NMR, respectively, and 13 of them were new compounds. Friedelin ring triterpenes are a very important class, and so far, these compounds were reported to display a lot of biological activity. For example, 28,30-dihydroxyfriedelan-3-one showed good anti-tumor activity against P388 [4].

*Euonymus hederaceus* (Celastraceae) is a scramble shrub cultivated as an ornamental or hedge plant and widely distributed in Anhui, Jiangsu, Zhejiang, Jiangxi, Fujian, Hunan, Guangdong, and Guangxi provinces in China and Taiwan. Its decoctions are reputed in traditional medicine for their antibiotic and anti-tumor properties [5]. *Euonymus hederaceus* was investigated for the first time in our group, and five known friedelane triterpenes: 3-friedelone, 28-hydroxyfriedelan-3-one (canophyllal), 28-hydroxy-3-friedelanone (canophyllol), 30-hydroxy-3-friedelanone, 29-hydroxy-3-friedelanone and three olean-type triterpenoids including 3β-methoxyolean-11-oxo-18-ene, olean-12-ene-3,11-dione and 28-hydroxyolean-12-ene-3,11-dione were separated and their structures elucidated [6]. We report here the isolation and structure elucidation of a new triterpene by a combination of NMR techniques, including ^1^H-NMR, ^13^C-NMR, DEPT135^0^, ^1^H-^1^HCOSY, HMQC and HMBC.

## Results and Discussion

**Figure 1:**
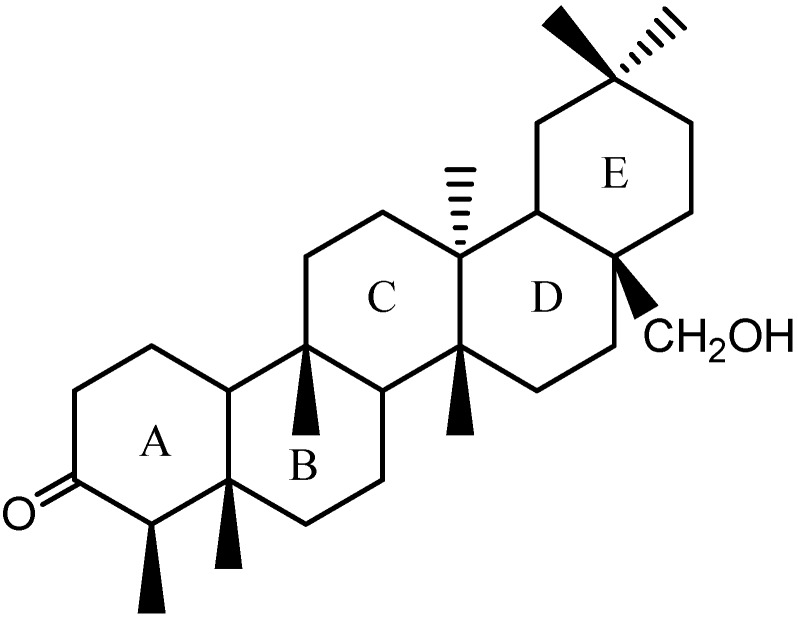
The structure of 28-hydroxy-friedelan-3-one.

**Figure 2:**
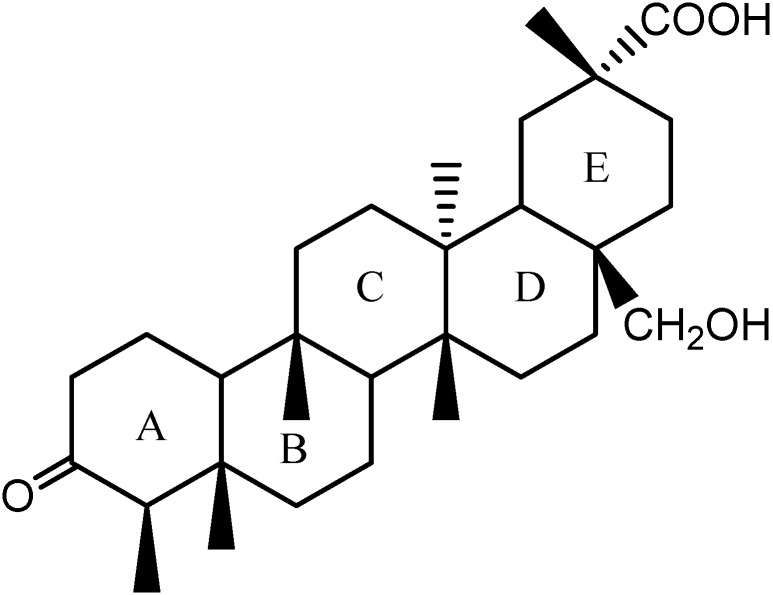
The structure of compound **1**.

Compound **1**: acicular crystals, m.p. 294-296 ^o^C, 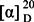: -8.016 (MeOH). Its molecular formula was deduced to be C_30_H_48_O_4_ (ESI-MS *m/z*: 473.3629 [M+H]^+^, calcd. 473.3625). The IR spectrum showed absorptions at υ_max_ 3,426, 1,718 and 1,707 cm^−1^, compatible with the presence of hydroxyl (OH) and two carbonyl functionalities, respectively. The ^1^H-NMR (C_5_D_5_N) spectra exhibited 46 protons, while the ^13^C-NMR and DEPT135^0^ spectra showed a total of 30 carbon signals: six primary carbons, twelve secondary carbons, four tertiary carbons and eight quaternary carbons, which were consistent with the characteristic of triterpene skeleton. Six methyl group signals at *δ* 0.66, 0.79, 1.04, 1.44, 1.45 (s, 3H each) and 0.95 (d, 3H) were observed in the ^1^H-NMR spectrum, in combination with six carbon signals at *δ* 14.6, 18.5, 15.7, 18.7, 33.0, and 7.2 (Table 1) in the ^13^C-NMR spectrum, which exhibited one methyl less than that of 28-hydroxy-friedelan-3-one. The HMBC correlations (Table 1) for the signals H-2 (*δ* 2.20), H-23 (*δ* 0.95), and H-4 (*δ* 2.12) with carbonyl carbon at *δ* 211.8 and for the signals H-28 (*δ* 3.89, 3.94) with C16, C22, and C18 were consistent with a 28-hydroxy-3-one type friedelane triterpene. Long-range correlations (HMBC) observed for the signals H-19 (*δ* 1.60), H-30 (*δ* 1.45), and H-21 (*δ* 1.77) with carbonyl carbon at *δ* 181.0 suggested the presence of another carbonyl group at C29, in agreement with the absorption at υ_max_ 1,707 cm^−1^ revealed by the IR spectrum. Comparing with the carbon spectrum with those of 28-hydroxy-friedelan-3-one (Figure 1) and 2-hydroxy-3-oxo-friedelan-29-oic acid [7,8] as well as 2D data (Table 1), differences were also observed: specifically a downfield shift at C20 (*δ* 40.8, for compound **1**; *δ* 28.1, for 28-hydroxy-friedelan-3-one) and C29 (*δ* 181.0, for compound **1**; *δ* 32.9, for 28-hydroxy- friedelan-3-one). Therefore, compound **1** was identified as 28-hydroxyfriedelan-3-one-29-oic acid (Figure 2).

**Figure 3:**
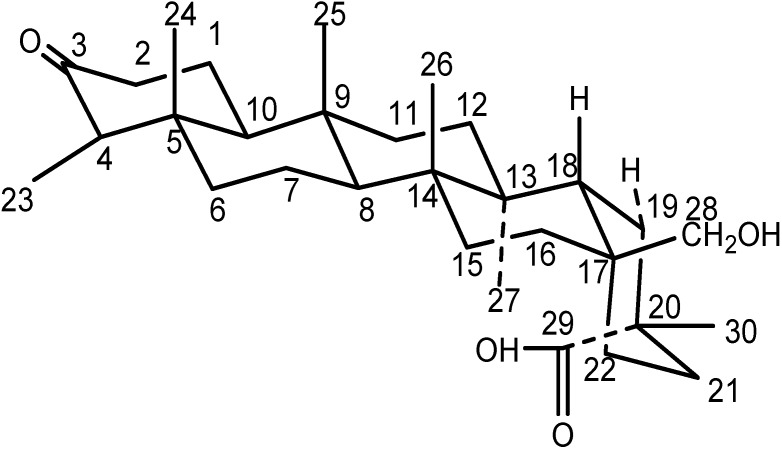
The chair-chair conformation of compound **1**.

As reported, the D and E-rings adopted boat-boat conformation for 3-friedelin, because the repulsion between C29 and C27 is very strong [9]. However, when C29 was oxidized to a carboxylic acid or aldehyde, the D and E-ring would favor chair-chair conformation due to release of the repulsion [4,10]. So it was speculated that rings D/E in compound **1** adopted a chair-chair conformation (Figure 3).

**Table 1:** 1D NMR and 2D NMR data for compound **1**.

Position	*δ_C_*			Compound 1	
	Compound 1 in pyridine-D_5_	28-hydroxy-friedelan-3-one	2-hydroxy-3-oxo-friedelan-29-oic acid	HMQC (*δ_H_*)	HMBC
C1	22.4	22.3	32.5	1.50(m), 1.73(m)	/
C2	41.5	41.5	75.0	2.20 (m), 2.38 (m)	/
C3	211.8	212.6	212.4	/	H-2,4,24
C4	57.8	58.1	55.6	2.12 (q, J=6.8)	H-23,24
C5	41.9	42.1	43.0	/	H-4,10,23,24
C6	41.1	41.2	41.2	1.10 (m), 1.54 (m)	H-24
C7	18.4	18.2	18.2	1.23 (m)	H-8
C8	50.4	52.4	50.8	1.39 (m)	H-25,26
C9	37.6	37.4	37.4	/	H-8,10,25
C10	59.2	59.4	56.8	1.40 (m)	H-24,25
C11	35.5	35.4	35.3	1.30 (m)	H-25
C12	29.7	30.1	29.5	1.80 (m)	H-27
C13	39.5	39.3	39.3	/	H-11,12,18
					H-27
C14	39.4	38.1	39.3	/	H-7,12,18,26 H-27
C15	29.1	31.2	29.4	1.41 (m)	H-26
C16	31.4	29.1	36.1	1.52 (m), 2.44 (m)	H-28
C17	35.6	35.1	30.1	/	H-18
C18	40.3	39.4	44.2	1.89 (m)	H-28
C19	30.3	34.5	30.3	1.60 (m), 2.62 (m)	H-30
C20	40.8	28.1	40.4	/	H-18,22,30
C21	31.1	31.4	29.7	1.77 (m), 2.82 (m)	/
C22	32.1	33.3	36.7	2.44 (m), 1.79 (m)	H-28
C23	7.2	6.8	6.6	0.95 (d, J 6.8)	H-4,5
C24	14.6	14.7	14.7	0.66 (s)	H-4
C25	18.5	18.1	18.5	0.79 (s)	H-8,10
C26	15.7	19.2	16.4	1.04 (s)	/
C27	18.7	19.1	18.1	1.44 (s)	H-18
C28	69.4	70.0	31.9	3.89,3.93(dd, J10.4)	H-18
C29	181.0	32.9	184.4	/	H-19,21,30
C30	33.0	34.3	31.4	1.45 (s)	H-21

## Experimental

### General

The IR spectrum was recorded in KBr pellets on a Nicolet NEXUS-470 FT-IR spectrometer. NMR spectra were recorded on a Bruker Avance DMX 500 NMR Instrument (Bruker Analytik, GmbH, Germany). The chemical shift values are given in ppm using pyridine-D_5_ as solvent and TMS as the internal standard. Mass spectra were performed on an Apex III (7.0 Tesla) Fourier transformation ion cyclotron resonance mass spectrometer (FT-ICRMS) equipped with electrospray ionization source (ESI) (Bruker, Billerica, MA, USA).

### Plant material

The whole plant of *Euonymus hederaceus* was collected in Jiulong Mountain, Suichang County, Zhejiang Province, P. R. China in October 2003, and identified by Dr. Haitong Wan (Zhejiang Chinese Medical University, Hangzhou, P. R. China.). A voucher specimen was deposited in the College of Agriculture and Biotechnology, Zhejiang University, Hangzhou, P. R. China.

### Extraction and isolation

The shade-dried, powdered root barks, stem barks and leaves of *Euonymus hederaceus* (5.0 kg) were extracted three times with 95% EtOH (20 L) at room temperature for seven days. After removal of the solvent *in vacuo*, the extract was dissolved in H_2_O (0.5 L) and extracted with EtOAc (2 L). The concentrated EtOAc extract (75 g) was subjected to column chromatography (CC) on silica gel, eluting with petroleum ether and increasing proportions of EtOAc. The eluate with 1:9 (petroleum ether/EtOAc) give the new pure compound **1** (10 mg).

## Conclusions

A new friedelane type triterpene was isolated from *Euonymus hederaceus*, its structure was determined to be 28-hydroxy-friedelan-3-one-29-oic acid by spectroscopic methods, including FT-ICRMS and NMR experiments, in combination to the comparison with known compounds. In addition, its stereo structure was proposed on the basis of the reference data.

## References

[1] Wang K.W., Sun H.X., Wu B., Pan Y.J. (2005). Two Novel Olean Triterpenoids from *Celastrus hypoleucus*. Helv. Chim. Acta.

[2] Wang K.W., Mao J.S., Tai Y.P., Pan Y.J. (2006). Novel skeleton terpenes from *Celastrus hypoleucus* with anti-tumor activities. Bioorg. Med. Chem. Lett..

[3] Wang K.W., Zhang H., Pan Y.J. (2007). Novel Triterpenoids from *Microtropis triflora* with Antitumor Activities. Helv. Chim. Acta.

[4] Nozaki H., Suzuki H., Lee K., Mephail A. (1982). Structure and stereochemistry of maytenfolic acid and maytenfoliol, two new antileukemic triterpenes from *Maytenus diversifolia*: *X*-ray crystal structures. J. Chem. Soc..

[5] Chang Z.F., Lu G.P., Wei J., Song F.X. (1996). Chinese medicinal plant *Celastraceae* properties preliminary processing. Chin. J. Inf. Trad. Chin. Med..

[6] Ren W.L., Hu H.J., Pan Y.J. (2006). Studies on olean-type triterpenoids of *Euonymus hederaceus*. J. Zhejiang Univ. (Science Edition)..

[7] Hongquan D., Yoshihisa T., Hiroshi M., Yasukazu O., Takao T., Jia Y., Li D. (2000). Triterpenoids from *Tripterygium wilford II*. Phytochemistry.

[8] Patra A., Chaudhuri S.K., Rübgger H. (1990). Complete ^13^C and ^1^H spectral assignments of friedelin by inadequate and heteronuclear (^13^C-^1^H) correlation experiments. J. Indian Chem. Soc..

[9] Mariano M.V., Miguel M.C., Cristina S.V., Lydia R.H., Pedro J.N. (1988). Terpenoids from *Mortonia diffusa*. J. Nat. Prod..

[10] Gottlieb H.E., Ramaiah P., Lavie A.D. (1985). ^13^C NMR signal assignment of friedelin and 3-hydroxyfriedelan-2-one. Magn. Reson. Chem..

